# Opportunities and Pitfalls in Applying Emotion Recognition Software for Persons With a Visual Impairment: Simulated Real Life Conversations

**DOI:** 10.2196/13722

**Published:** 2019-11-21

**Authors:** Hendrik Buimer, Renske Schellens, Tjerk Kostelijk, Abdellatif Nemri, Yan Zhao, Thea Van der Geest, Richard Van Wezel

**Affiliations:** 1 Department of Biophysics Radboud University Nijmegen Netherlands; 2 Department of Biomedical Signals and Systems University of Twente Enschede Netherlands; 3 VicarVision Amsterdam Netherlands; 4 Center IT + Media Hogeschool van Arnhem en Nijmegen University of Applied Sciences Arnhem Netherlands

**Keywords:** visual impairment, emotion recognition, tactile, social interaction

## Abstract

**Background:**

A large part of the communication cues exchanged between persons is nonverbal. Persons with a visual impairment are often unable to perceive these cues, such as gestures or facial expression of emotions. In a previous study, we have determined that visually impaired persons can increase their ability to recognize facial expressions of emotions from validated pictures and videos by using an emotion recognition system that signals vibrotactile cues associated with one of the six basic emotions.

**Objective:**

The aim of this study was to determine whether the previously tested emotion recognition system worked equally well in realistic situations and under controlled laboratory conditions.

**Methods:**

The emotion recognition system consists of a camera mounted on spectacles, a tablet running facial emotion recognition software, and a waist belt with vibrotactile stimulators to provide haptic feedback representing Ekman’s six universal emotions. A total of 8 visually impaired persons (4 females and 4 males; mean age 46.75 years, age range 28-66 years) participated in two training sessions followed by one experimental session. During the experiment, participants engaged in two 15 minute conversations, in one of which they wore the emotion recognition system. To conclude the study, exit interviews were conducted to assess the experiences of the participants. Due to technical issues with the registration of the emotion recognition software, only 6 participants were included in the video analysis.

**Results:**

We found that participants were quickly able to learn, distinguish, and remember vibrotactile signals associated with the six emotions. A total of 4 participants felt that they were able to use the vibrotactile signals in the conversation. Moreover, 5 out of the 6 participants had no difficulties in keeping the camera focused on the conversation partner. The emotion recognition was very accurate in detecting happiness but performed unsatisfactorily in recognizing the other five universal emotions.

**Conclusions:**

The system requires some essential improvements in performance and wearability before it is ready to support visually impaired persons in their daily life interactions. Nevertheless, the participants saw potential in the system as an assistive technology, assuming their user requirements can be met.

## Introduction

### Background

A large number of communication cues exchanged between persons are nonverbal (eg, gestures, facial expressions, and gaze direction). As a result, the inability to perceive these cues leads to a loss of communication effectiveness. Not perceiving these nonverbal cues is particularly present in persons who are blind or visually impaired (hereafter referred to as visually impaired persons) and can lead to feelings of exclusion [1,2]. Previous inventory studies of assistive technology needs [3,4] found that a need among the community of visually impaired persons still exists for a solution that makes nonverbal signals accessible.

To convey such information and make it accessible for visually impaired persons, visual information can be *translated* into auditory or tactile cues, which is the foundation of sensory substitution devices (SSDs). The SSD developed by Bach-y-Rita [5], often considered as the first SSD, translated a video feed into a grid of vibration motors attached to the back of a chair. Visually impaired persons trained to use this system were able to pick up an object. More recently, the vOICe system (which translated a video with very high contrast directly into auditory cues) and a Tongue Display Unit were developed (which translated video into electric signals on the tongue) [6-8]. However, these systems rely largely on senses and body parts that are crucial for interpersonal communication, that is, mouth and ears. In the last years, various researchers have acknowledged the issue of nonverbal information and worked toward a range of sensory-substitution designs: information such as interpersonal distance, location of others, facial expressions of emotions, and gestures was conveyed using various vibrotactile devices, including the back of a chair [9,10], a glove [11], and a vibrotactile belt [12,13].

In addition, recent advances in machine learning and computer vision technologies, which improved the ability of computers to recognize patterns from images, allow for very specific recognition tasks. One of the opportunities that arose from these advancements is the possibility to train a computer to recognize faces and facial expressions from a video. This idea was pioneered by Bartlett et al in a collaboration with Paul Ekman [14], leading to effective emotion recognition software. Among its numerous applications, such software is particularly interesting for people who are unable to detect and interpret facial expressions of emotions, such as visually impaired persons. Given the need from the community and the potential of emotion recognition software in meeting these needs, we investigated to what extent such emotion recognition software can support visually impaired persons in their daily lives.

Previously, we presented a wearable system for facial emotion recognition consisting of a head-mounted camera and a vibrating belt [15]. It was designed to help visually impaired persons perceive facial expressions of the 6 universal emotions [16]. Via a tiny camera attached to standard spectacles, pointing in the direction where the wearer is looking, a continuous video stream was available for processing. To determine which emotions were expressed, FaceReader facial expression recognition software was applied. This software can be used to detect Ekman’s universal emotions from pictures and videos [16-18]. In this study, FaceReader was applied to analyze the video stream, originating from the video camera, in real time. Once a face is detected, the software attempts to create a map of the face including almost 500 facial landmarks. If a face muscle movement is detected, the deviation from a baseline neutral face is recognized as a facial expression and then classified as an emotion. Depending on the occlusion of the faces, either a standard model (called 3D Active Appearance Model) or proprietary deep neural networks are used to assign a score to the intensity of the facial expression (emotion). Once an expression was detected as being 1 of the 6 universal emotions, vibrotactile signals on a waist belt conveyed this information to the wearer of the system. Previous research has shown that vibrotactile signals were interpretable and usable in cognitively and physically demanding tasks such as flying a helicopter or steering a boat [19]. By signaling emotions through haptic feedback at the waist during social interactions, the ears of the users remained free to engage in a conversation.

This system was previously tested with visually impaired persons in a controlled laboratory setting [15], with pictures from the Warsaw Set of Emotional Facial Expression Pictures (WSEFEP) [20] and videos from the Amsterdam Dynamic Facial Expression Set (ADFES) [21]. Such validated sets of images and videos of actors showing strong emotional expressions in controlled laboratory conditions play an important role in the training and development of emotion recognition software but do not entirely resemble realistic facial expressions [22]. The results of our first study were promising, as the FaceReader software reached high recognition scores and users were able to easily learn how to interpret and use the vibrotactile signals from the system [15]. That the system worked well under controlled laboratory lighting in classifying very expressive facial expressions was expected, as emotion recognition software often achieved high recognition scores when tested with validated sets [18,23]. On validated sets such as the ADFES and WSEFEP, FaceReader was known to achieve recognition rates of 89% and 88%, respectively [17]. However, these sets are often acquired during photoshoots with actors who express strong emotional states under optimal lighting conditions. Facial expressions in real life are often much subtler than those found in the validated sets, and lighting conditions also differ between real life and controlled conditions [22,24,25]. As a result, we cannot be certain that the recognition scores achieved from these validated sets can be replicated once such software is faced with genuine facial expressions of emotions, expressed in natural conversations. Thus, to determine whether the technology applied is useful for this purpose, its performance must be further tested under real-world conditions [26].

### Objectives

To investigate whether the system is usable and useful during realistic conversations, we aimed to examine the following questions: (1) were visually impaired persons able to keep the camera focused on the face of the conversation partner?; (2) did the software reach a satisfactory recognition rate when confronted with an unstable video stream and suboptimal lighting conditions?; (3) were visually impaired persons able to interpret the emotion cues while engaging in a meaningful conversation?; and (4) did the system live up to prospective users´ expectations, and did they believe they could use such technologies in their daily lives?

## Methods

### Ethical Approval

The study was designed in accordance with the Declaration of Helsinki and approved by the ethical committee of the Faculty of Electrical Engineering, Mathematics and Computer Science of the University of Twente, Enschede.

### Participants

A total of 8 visually impaired persons (4 females and 4 males; mean age 46.75 years, age range 28-66 years) were included in the study. Moreover, 4 of the participants were visually impaired from birth, whereas the others became visually impaired later in life but had experience with vision for at least 10 years. All participants reported difficulties with the recognition of facial expressions and did not suffer from any other cognitive or sensory impairments (Table 1).

**Table 1:** Participants.

ID	Age (years)	Gender	Visual impairment	Sight	10 or more years of vision	Emotion logs available^a^
1	28	Male	Neurological damage to eye nerves	Tunnel	No	Yes
2^b^	28	Male	Persistent fetal vasculature	None	No	No
3^b^	66	Male	Retinitis pigmentosa	None	No	No
4	59	Female	Retinitis pigmentosa, glaucoma, and cataract	None	Yes	Yes
5	47	Male	Cone dystrophy	Peripheral	Yes	Yes
6	64	Female	Congenital rubella syndrome	None	No	Yes
7	50	Female	Retinitis pigmentosa	None	Yes	Yes
8	32	Female	Aniridia	<5%	Yes	Yes

^a^See the section Data Collection and Analysis.

^b^No emotion logs were collected during the experiment.

### Apparatus

The experiments were conducted at an observation room at the HAN University of Applied Sciences (Arnhem, The Netherlands), where it was possible to discreetly observe the experiment from an adjacent viewing room and record with 3 different cameras and 2 microphones for postanalysis.

**Figure 1:**
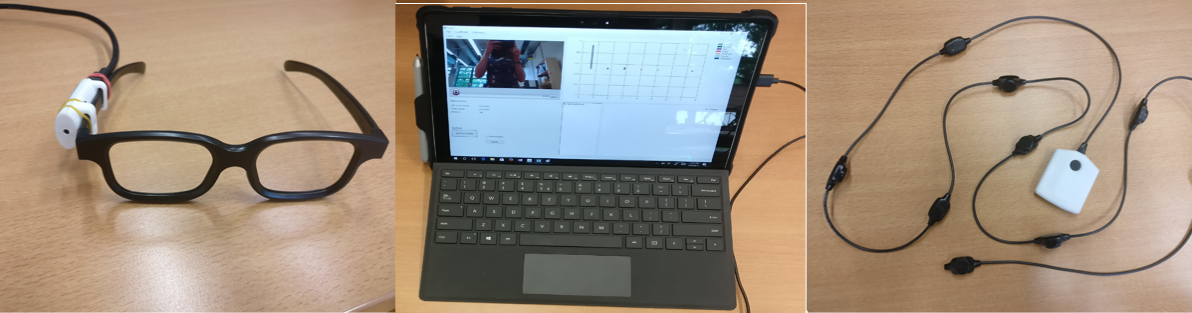
Schematic overview of the used system.

The emotion recognition system used in the study consisted of a small USB camera clipped on spectacles; a Microsoft Surface Pro 4 tablet running the FaceReader 6 SDK emotion recognition software (running the general face model); and a chain of 9 vibrotactile stimulators attached to a Velcro belt, 6 of which were used (see Figure 1). To ensure steady connections between the components, cable connections were used.

**Figure 2:**
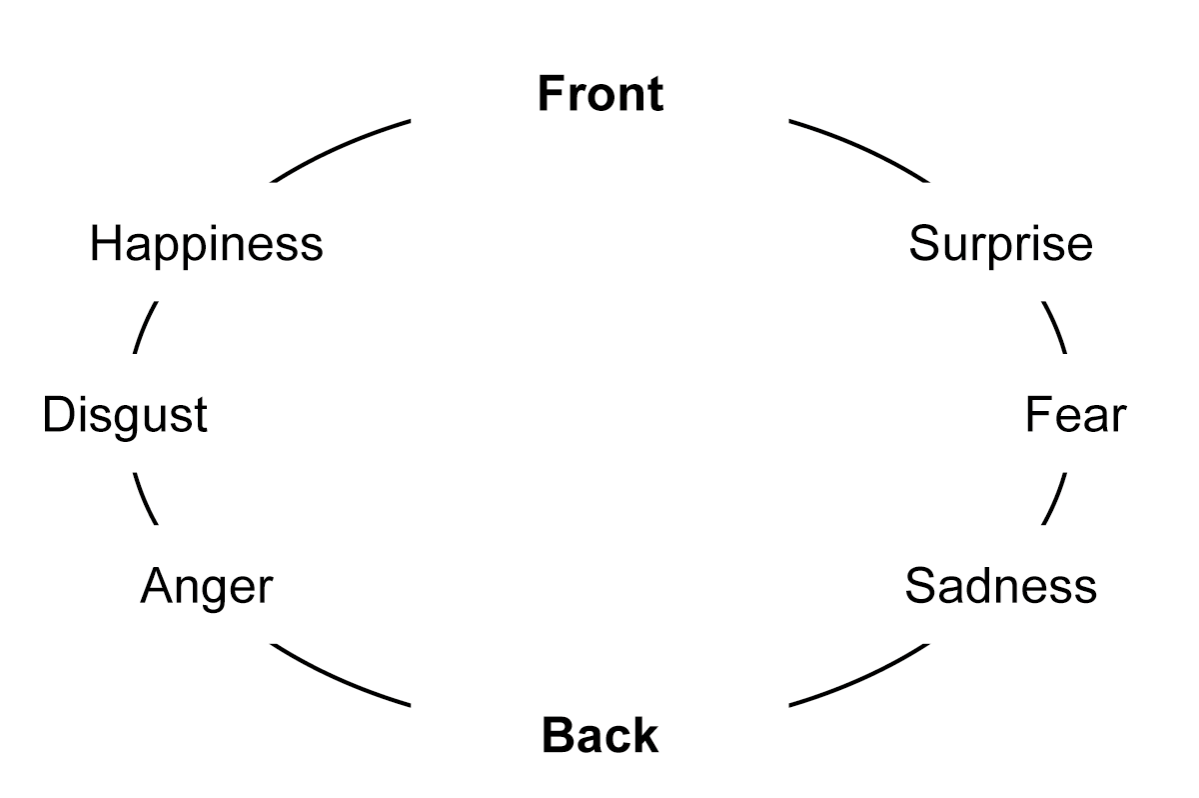
Emotion mapping. The mapping of Ekman’s universal emotions on the waist band.

The FaceReader system classified facial expressions into 1 of the 6 of Ekman’s universal emotions: happiness, sadness, anger, fear, surprise, and disgust [16]. When an emotion is recognized, feedback was given to the participant via the vibrotactile stimulators worn around the waist. Each emotion was assigned to a single vibrotactile stimulator (Figure 2). The decision to use a belt with vibrotactile stimulators and let it be worn on the waist had several reasons. First, the tactile modality was chosen because the use of the auditory modality is undesirable during social interactions, in which auditory information is very important for visually impaired persons and should, therefore, not be interfered with. Second, the tactile resolution of the waist is enough to place 6 vibrotactile stimulators without the risk of users being unable to distinguish them. A belt can be worn unobtrusively underneath clothing and wearing it at the waist means that the system does not interfere with the conversations in the sense that it keeps your eyes, ears, and hands free.

Vibrotactile stimulators were activated when the associated emotion was detected above a certain threshold of certainty, after which the vibration strength indicated the certainty that the software had detected an emotion. The standard threshold to initiate a vibrating pulse was 0.3 (on the FaceReader scale from 0 to 1), with 2 exceptions. In pretests with an actor, the FaceReader software was more sensitive to some emotions than to others. For example, when the actor was concentrating on listening and looking interested, his facial expression was at times classified as angry. Therefore, the threshold for the emotion anger was raised to 0.6. A threshold of 0.7 was chosen for the emotion happiness as it was rapidly detected even if the expression was barely visible.

### Procedure

The study consisted of 4 different phases: initial training, refresher training, the experiment, and an exit interview.

First, the participants were trained to interpret the vibrotactile signals during 2 separate training sessions (Table 2). The first session took place about 2 weeks before the experiment. After a brief introduction to the tactile mapping around the waist, the training included application of 4 sets of 36 vibrotactile stimuli. After each stimulus, the participant was asked to indicate which emotion was signaled. These stimuli were initially accompanied with matching auditory cues that matched the vibrotactile feedback. The number of audio-accompanied stimuli steadily decreased (100%, 50%, 33%, and 0%), resulting in a full dependency on vibrotactile signals in the fourth set. In these sets, each emotion was equally represented. The fifth and last set included only vibrotactile feedback. The minimal number of stimuli presented in this set was 20, whereas the maximum number of stimuli was set at 96. It was ended when participants achieved a 95% success rate over the last 20 stimuli that were presented to them. The initial training phase lasted approximately 65 min.

The refresher training session took place just before the experiment and consisted of 3 sets. The first 2 sets included 24 stimuli in which the emotions were equally represented. The first set was fully accompanied by audio, whereas the second set included only vibrotactile feedback. The third set was the same as the final set of the initial training. It included 96 vibrotactile-only stimuli and was ended when participant achieved a 95% correct rate in the last 20 stimuli presented to them. The refresher training had an average duration of approximately 30 min.

After training was completed, the participants engaged in 2 conversations with an actor for approximately 15 min. The conversations were set up as a mock job interview in which the actor played the role of the director of a fictional company. The actor and the participant were seated at a table approximately 1.5 m opposite of each other.

The first conversation was colloquial, whereas the second was more formal. This was done to put the participants at ease and because we expected that the quantity and type of facial expressions of emotions might differ between the two.

The actor followed a similar structure for all conversations with the 8 participants. He was instructed not to exaggerate any facial expressions to keep the conversations as realistic as possible. A total of 4 participants wore the system in the first conversation, whereas the remaining 4 wore it during the second conversation.

After the experimental session, a semistructured exit interview was conducted to allow participants to share their thoughts and ideas about the system. It addressed various themes associated with system functionality, user experience, and technology acceptance (see Table 3).

**Table 2:** Stimuli distribution. Overview of the number of stimuli that were presented to the participants in each training session and the percentage of audio-accompanied stimuli.

Session	Total number of stimuli (percentage audio accompanied stimuli), n (%)				
1	36 (100)	36 (50)	36 (33)	36 (0)	20-96 (0)^a^
2	24 (100)	24 (0)	20-96 (0)^a^	—^b^	—

^a^Ended after a 95% correct score in the last 20 stimuli.

^b^The second session included only three sets of stimuli.

**Table 3:** Exit interview topics. An overview of the topics discussed in the exit interview, the associated questions, and the subthemes of interest.

Topic, exit interview question		Subthemes
**Experience**		
	How was your experience using the system?	Accuracy, usefulness, feedback, fun of use, and ease of use
	What was the value of the system during the conversation?	Additional value, distraction from the conversation, and interpretation of the system
**Potential utility**		
	Would you like to use the system in your daily life?	System usage, situations of use, and recommendation to others
	Imagine you have a job interview next week and we will lend you the system. Would you use it during the job interview?	Usage in job interview
**Adjustments to the system**		
	If you are the manager of the team that develops this system, what do you think they should tackle first?	Improvements of the system and additions to the system
**Social acceptance**		
	How do you expect people in your surroundings (eg, family, friends, and colleagues) will react to you using a system like this?	Reaction of surroundings and introduction of the system

### Data Collection and Analysis

After the experimental sessions, 5 sets of data were acquired. First, the training data were collected and analyzed by tallying the number of correct and wrong answers for each set of stimuli. Second, to determine the performance of the emotion recognition software, video recordings and emotion logs were saved and reviewed. The actor and the participant were filmed from 3 different camera positions (1 ceiling camera aimed at the participant, and 1 stationary camera aimed at the participant, and 1 ceiling camera focused on the actor). The emotion logs kept a record of the emotion recognition software by saving timestamps together with the strength of a detected emotion (a value between 0 and 1; an example of such a log can be found in Table 4). Each time the software detected an emotion above threshold for at least 5 timestamps within a time span of 1 second, a fragment was labeled as an emotion signal. Due to technical issues, the emotion logs of only 6 out of the 8 experiments were analyzable.

**Table 4:** Example of an emotion log. The emotion log provides a value between 0 and 1 for each emotion at each timestamp (hours [hh]:minutes [mm]:seconds[ss]:milliseconds[ms]).

hh	mm	ss	ms	Neutral	Happiness	Sadness	Anger	Surprise	Fear	Disgust
14	1	24	226	0.848577	0.261171	0.045551	0.037921	0.066328	0.101152	0.034363
14	1	24	279	0.853517	0.246223	0.052067	0.039493	0.064576	0.089338	0.034737
14	1	24	354	0.860102	0.227184	0.061851	0.045071	0.060259	0.075662	0.033203
14	1	24	439	0.868132	0.206724	0.068306	0.054500	0.054876	0.063745	0.032779
14	1	24	550	0.875653	0.181231	0.069503	0.060954	0.048684	0.050882	0.034567
14	1	24	617	0.866770	0.157769	0.065179	0.063756	0.041683	0.042549	0.050491
14	1	24	673	0.850679	0.149551	0.064955	0.064503	0.038547	0.039288	0.058762
14	1	24	742	0.834428	0.144484	0.065088	0.065431	0.036603	0.038934	0.061535
14	1	24	798	0.807851	0.137719	0.065118	0.067514	0.033447	0.040757	0.061940

The third dataset consisted of the relationship between the emotion log fragments and the video recordings. For every instance FaceReader recognized an emotion, as was derived from the emotion logs, a video fragment was created, resulting in 166 video fragments that required annotation across all videos. A total of 2 independent coders then analyzed each fragment to determine if they recognized a facial expression of any of the 6 universal emotions (happiness, sadness, anger, fear, surprise, and disgust) and annotated the video fragments accordingly. In cases where the coders did not identify 1 of these emotions (be it seeing another emotion or seeing no emotion at all), the fragment was coded as “*none/other*.” Eventual disagreements were discussed in person to reach consensus.

To analyze how well the software detected emotions, a fourth set was used. This set consisted of a sample of every third minute that was extracted from the videos. This resulted in a total of 29 min of video that were analyzed for facial expressions of emotions, independent of the emotion logs. A first coder analyzed the videos and identified fragments in which facial expressions of emotions were thought to be visible. A second coder analyzed the same sample of randomly selected videos while seeing the selected fragments by the first coder. The second coder checked whether there was an agreement with the expressions identified by the first coder or if new expression were to be added. Again, any disagreements were discussed to reach consensus. Finally, the fragments in which facial expressions were detected by the 2 coders were compared with the emotion logs to analyze how well the system detected facial expressions of emotions during the conversations.

The fifth and final set were the exit interview transcriptions. The exit interviews were recorded and transcribed verbatim, which resulted in 70 pages of text. A content analysis was performed to extract the most important information fragments from the transcriptions. Codes were assigned to all fragments, based on the question to which the fragments provided an answer. To structure the answers, the subthemes mentioned in Table 3 were assigned to the answers.

## Results

### Learn to Interpret Vibrotactile Signals

Both training sessions successfully taught participants how to interpret the vibrotactile signals. Participants were quickly able to correctly identify most stimuli. The average performance gradually improved over each set of stimuli. Moreover, 6 out of the 8 participants achieved perfect scores in the final set (Table 5).

The refresher session, right before the experiment, showed even better results, ending with a perfect recognition rate for all participants in the third and final set (Table 6).

It can be concluded that during the training session, most participants were able to learn how to identify the correct emotions from the vibrotactile signals.

**Table 5:** Number of errors during the initial training. The table shows the numbers of errors of all participants during the initial training session.

ID	Set 1	Set 2		Set 3		Set 4	Set 5	Total
	A^a^ (n=36)	A (n=18)	T^b^ (n=18)	A (n=12)	T (n=24)	T (n=36)	T (n=20-96)	
1	3	0	4	0	0	1	0	8
2^c^	0	0	0	0	0	0	0	0
3^c^	10	3	0	1	0	1	7	22
4	0	0	0	0	1	0	0	1
5	0	0	0	0	0	0	0	0
6	0	0	0	0	0	0	0	0
7	1	0	2	0	0	0	0	3
8	0	0	1	1	0	1	2	5
Total	14	3	7	2	1	3	9	39

^a^A: auditory accompanied vibrotactile stimuli.

^b^T: tactile only stimuli.

^c^No emotion logs were available for this participant in the experiment.

**Table 6:** Number of errors during the refresher training session. The table shows the number of errors of all participants during the refresher training session.

ID	Set 1, A^a^ (n=24)	Set 2, T^b^ (n=24)	Set 3, T (n=20-96)	Total
1	1	0	0	1
2^c^	0	1	0	1
3^c^	6	0	0	6
4	0	0	0	0
5	0	0	0	0
6	0	0	0	0
7	0	0	0	0
8	2	0	0	2
Total	9	1	0	10

^a^A: auditory accompanied vibrotactile stimuli.

^b^T: tactile only stimuli.

^c^No emotion logs were available for this participant in the experiment.

### Camera Pointing

A prerequisite for the emotion recognition system is that participants can focus the camera on the face of the conversation partner. Generally, the participants were able to focus the camera on the actor who sat on the other side of the table. In 5 out of the 6 videos analyzed, the face of the actor was out of sight for only a couple of seconds out of the approximately 15 min that the conversation lasted. Moreover, 1 participant was an exception to this rule. This participant tended to slightly lift her head upward. As a result, the face of the actor was out of the field of view of the camera many times, rendering the emotion recognition software useless in our context.

### Emotion Recognition Performance

#### Agreement With Software-Detected Emotions

This analysis was based on the video fragments in which the software had detected an emotion. A total of 2 coders annotated 166 fragments for which the emotion log values exceeded threshold levels for both emotion value and duration. Initially, the coders disagreed 30 times, but mutual consensus was achieved after reviewing the fragments together.

The agreement between the codes assigned by the coders and emotion recognition software was poor. In only 54 out of the 166 fragments, the coders and FaceReader classified the same universal emotions (Table 7).

In 13 cases the coders agreed upon seeing 1 of the 6 universal emotions but differed from the emotion detected by the software. For most fragments (99), however, the coders disagreed with the software in the sense that the software detected a universal emotion, whereas the coders did not. In this analysis, the coders and the software showed most agreement on the emotion happiness, whereas the performance for other emotions is far worse. It can be concluded that the coders disagreed with the signals that FaceReader conveyed for 5 out of the 6 emotions. Only when it came to the signaling of happiness, the coders largely agreed with the software.

**Table 7:** Crosstabs of agreement between coders and software. The table shows a tally of the number of time the coders and FaceReader classified a fragment as a particular emotion. The diagonal shows the number of times that the coders and FaceReader classified a fragment as the same emotion.

Facereader	Coders							
	Anger	Disgust	Happiness	Sadness	Fear	Surprise	None/other	Total
Anger	*2*	0	0	0	0	0	8	10
Disgust	1	0	0	0	0	0	12	13
Happiness	0	0	*42*	0	0	1	3	46
Sadness	1	0	2	*4*	0	1	41	49
Fear	0	0	2	0	0	1	4	7
Surprise	0	0	4	0	0	*6*	31	41
Total	4	0	50	4	0	9	99	166

#### System Performance to Detect Facial Expressions of Emotions

To determine how well the system derived facial expressions from the actor, we checked the random sample of every third minute. In this time, the 2 coders detected 72 facial expressions of basic emotions and another 12 expressions not necessarily being associated with 1 of Ekman´s emotions. The software detected only 44 in the same time frame. In 20 cases there was an agreement between the human coders and the system detection. A total of 24 times the human coders and FaceReader disagreed about emotions being expressed. The remaining 51 facial expressions of universal emotions that were detected by the coders (31 instances of happiness and 20 instances of surprise) were not recognized by FaceReader (Table 8).

These results show that in the random sample of videos, mostly happiness and surprise were expressed according to the coders. In the 44 cases where the software did detect an emotion, the coders disagreed in almost half of the cases. The only exception seemed to be happiness, which, if it was detected, was agreed upon in 17 of 19 cases. However, the coders detected many more instances of happiness that the software was unable to detect. This was similar for facial expressions of surprise.

**Table 8:** Overview of the agreement between human coders and the software for the facial expressions detected. The table shows the classification of detected emotions.

Emotions	Agreement	Disagreement	Not detected by FaceReader^a^
Anger	0	5	0
Happiness	17	2	31
Disgust	0	2	0
Sadness	0	10	0
Fear	0	2	0
Surprise	3	3	20
Total	20	24	51

^a^The coders identified 11 other facial expressions that did not classify as 1 of the universal emotions.

#### User Experience

Besides the objective measures to analyze the technical performance of the system in a realistic condition, we asked the participants how was their experience using the system and whether they saw potential utility of the system in their daily lives.

How did persons perceive the vibrotactile cues during the conversation?:

There was no doubt about the transferred information. Once you know the location of emotions, the usage of the system is very convenient.

P2, male, aged 8 years, fully blind

A total of 4 participants even believed they were able to subconsciously interpret the vibrotactile signals:

I am consciously participating in the conversation while I am taking the feedback subconsciously into account, so the vibrations did not distract me, absolutely not.

P4, female, aged 59 years, fully blind

Moreover, 3 others found it difficult to focus their attention on the signals and struggled to interpret them. Participant 2 (male, aged 28 years, fully blind) felt that the prolonged stimuli really disrupted his ability to engage in the conversation with the actor. Participant 5 (male, aged 47 years, partially sighted) found it particularly difficult to interpret the signal while he was speaking himself, although he felt that the interpretation of “happiness” and “surprise” was easier than the other emotions as these occurred more frequently than other emotions. Indeed, during his conversation, happiness and surprise were conveyed quite often (7 and 5 times, respectively), although sadness was conveyed as often as surprise.

In addition, 3 participants did not realize that vibration strength was linked to the intensity of the expressed emotion and explicitly requested for such a functionality. Participant 1 (male, aged 28 years, partially sighted) suggested to use pulsating vibrations instead to convey intensity. Thus, to convey such information other methods are wished for.

A total of 7 participants were skeptical about the accuracy of the conveyed emotions, as these did not always correspond to the vocal cues and the atmosphere during the conversation. Participant 4 (female, aged 59 years, fully blind), for example, reported to have received signals of a disgusted facial expression, whereas she felt there was absolutely no reason to believe the actor looked disgusted. In the video analysis of her interview with the actor, no facial expressions of disgust were identified by the 2 coders, whereas there were 7 instances where the software conveyed disgust. Participant 1 (male, aged 28 years, partially sighted) felt that the system did consistently provide information at the moments it was most necessary. Moreover, 2 persons reported receiving only limited signals.

Despite the inaccuracy, 6 participants were convinced about the usefulness of the concept of an emotion recognition system. Participant 7 (female, aged 50 years, fully blind) believed such a system would especially be useful in the absence of vocal cues, where it would be the only source of information. In addition, confirmation of the emotions derived from vocal cues led to more confidence. Furthermore, 1 participant (P5, male, aged 47 years, partially sighted) mentioned that the system could be valuable while the user himself is talking, as feedback on emotional expressions of the conversation partner in such situations can make the user more confident about the story he or she is telling. On the downside, 2 persons reported that because of inaccuracies, the system caused more confusion than clarity. Participant 4 (female, aged 59 years, fully blind) mentioned that she believed she could detect faulty signals and therefore was not so much deterred by the errors. It could be that some participants are more willing to accept errors as these did not weigh up against the experienced usefulness of the system.

A total of 4 participants wanted to use the system in their daily life, whereas 3 others wished for more time with the system before they could decide. Moreover, 2 persons were convinced that the system, as it is, would be an added value to their life. In addition, 3 others believed that the system would have an added value after some adjustments.

Besides the obvious accuracy improvements, participants wished for improvements in the wearability of the system and requested additional functionalities. A total of 7 participants said they would like a more portable system, which could be achieved by going fully wireless. All but 1 participant requested for a camera that is integrated in the spectacles. The 1 participant who did not request for a camera integrated in spectacles (P5, male, aged 47 years, partially sighted) suggested that the camera should be clearly visible to make conversation partners aware they are being filmed. Moreover, 2 participants suggested to get rid of the spectacles completely and allow users to clip the camera anywhere on their clothing.

A total of 7 out of the 8 participants believed that a person recognition function would increase the usefulness of the system. Participant 1 (male, aged 28 years, partially sighted) even stated that emotion recognition and face recognition are inseparable, as in a busy environment you need to know who you look at before emotion recognition becomes relevant. Such technologies could also be applied to locate a specific person that you are looking for. Related to this, participant 2 (male, aged 28 years, fully blind) wished for a function to request for feedback from the system at any time to check whether the system is still on and focused after long periods of inactivity. Such a trigger was also suggested by participant 1 (male, aged 28 years, partially sighted), who wished for a trigger to switch the feedback on or off to avoid an information overload from the system.

## Discussion

### Principal Findings

The main objective of this study was to determine whether the system presented in an earlier study [15], which was successfully tested in laboratory conditions, would work in a realistic conversation situation. The study affirmed that a positive laboratory test does not necessarily mean that a system is ready for real-life usage [26]. Although the concept of our system seems to have potential, it requires essential improvements before it is ready to support visually impaired persons in real life.

We have confirmed findings from a previous study [5] and showed that it is very easy to learn how to interpret vibrotactile cues signaled with a haptic waist belt. That in itself is not a surprise, both older [5,27] and more recent [28,29] studies have shown that much more complex tactile patterns can be successfully learned. Furthermore, it was already known that healthy persons can interpret vibrotactile cues in demanding environments [30]. However, what is interesting is that we now have indications that users of the system with a visual impairment were able to use information conveyed through vibrotactile signals while they engaged in a conversation.

Most of the participants were able to keep the camera focused in the direction of the actor for most, if not all the, time. That means that the principle of putting a camera on spectacles will provide a sufficient camera feed for such computer vision analysis. Moreover, 1 person, however, aimed the camera above the actor for most of the time. Before using such a system, this person would require training on how to aim the camera to benefit from the system. Another option would be to redesign the camera and spectacles in such a way that the camera angle can be altered to match the wearer’s preference. This is a feature that some mobile eye trackers already have.

The accuracy of the software was not sufficient to convey all of Ekman’s [16] 6 universal emotions under our experimental conditions. Earlier studies on the performance of the software were conducted using validated sets composed of pictures including high-quality full-face pictures in ideal lighting conditions. In such conditions, the software used in the study achieved accuracy scores as high as 88% [17]. In our experiment, however, the software was confronted with suboptimal lighting conditions and unstable shots because of a moving camera and actor. In these conditions, the only emotion that was accurately recognized was happiness, which was also reported by the participants. This is if the emotion was recognized, as the coders detected 31 more instances of a facial expression of happiness that the software did not detect. The other 5 emotions were poorly recognized. As a result, the performance achieved in the experiment was too poor to be a support for visually impaired persons in real life.

It is unlikely that the system presented in the study would be used by the participants in their daily lives in its current form. Too many adjustments were suggested to believe that implementation would be easy (eg, emotion recognition accuracy, portability, person recognition, and on/off switch). Nevertheless, there is reason to believe that such a system might be used, once the required adjustments are adapted.

### Limitations

The emotions were not uniformly represented in the conversations. It is clear that the emotion happiness was far more often detected and conveyed via vibrotactile feedback than the other emotions. Therefore, it is difficult to draw definitive conclusions on the accuracy of the other emotions.

During the experiment, it was impossible to objectively measure if and how well participants were able to perceive the vibration signals. Therefore, we had to rely on the reports by the participants when it comes to the interpretation of signals.

We chose not to encode any other emotions than the 6 universal emotions. As a result, we cannot provide an overview of all the facial expressions of emotions that were expressed in the conversations. Thus, we cannot state which emotions are more often present in realistic conversations and could possibly be added to the emotion recognition software.

### Conclusions

For systems such as the one presented in this study, the main improvements to be made should be sought in the direction of both the wearability of the system and improvement of the emotion recognition software. The solution for wearability would be to make components of the system smaller (from tablet to smartphone) and by making all connections between system components wireless. To improve emotion recognition software, we suggest training with more ambiguous and subtle facial expressions under different lighting conditions. We do believe that if these concerns are taken care of, systems such as the one presented here might be of great benefit for visually impaired persons in their future daily social interactions.

## Abbreviations

ADFES: Amsterdam Dynamic Facial Expression Set
SSD: sensory substitution device
WSEFEP: Warsaw Set of Emotional Facial Expression Pictures
